# [Retracted] Sequential use of protein kinase inhibitors potentiates their toxicity to melanoma cells: A rationale to combine targeted drugs based on protein expression inhibition profiles

**DOI:** 10.3892/ijo.2024.5707

**Published:** 2024-11-18

**Authors:** Philippe G. Aftimos, Murielle Wiedig, Mireille Langouo Fontsa, Ahmad Awada, Ghanem Ghanem, Fabrice Journe

Int J Oncol 43: 919-926, 2013; DOI: 10.3892/ijo.2013.2008

Following the publication of this article, a concerned reader drew to the Editor's attention that there appeared to be the duplication of a pair of western blots in each of Figs. 4 and 6, with the possibility of the bands in question having been resized in one of these cases. After having conducted an internal investigation, the Editorial Office also determined that there was a further instance of duplication of western bands comparing between Figs. 2 and 4. In all cases, the bands that had been re-used were intended to show the results of differently performed experiments.

After assessing the issues that have come to light with regard to the duplications of western blots in this paper, in view of the apparently misassembled data that has been identified in each of Figs. 2, 4 and 6, the Editor of *International Journal of Oncology* has determined that this paper should be retracted from the publication on the grounds of a lack of confidence in the presented data. Upon contacting the authors, they accepted the decision to retract this paper. The Editor apologizes to the readership for any inconvenience caused, and we also thank the reader for bringing this matter to our attention.

